# Acyl-CoA:Diacylglycerol Acyltransferase 1 Expression Level in the Hematopoietic Compartment Impacts Inflammation in the Vascular Plaques of Atherosclerotic Mice

**DOI:** 10.1371/journal.pone.0156364

**Published:** 2016-05-25

**Authors:** Nemanja Vujic, Jess Porter Abate, Stefanie Schlager, Tovo David, Dagmar Kratky, Suneil K. Koliwad

**Affiliations:** 1 Institute of Molecular Biology and Biochemistry, Medical University of Graz, Graz, Austria; 2 Diabetes Center, University of California San Francisco, San Francisco, California, United States of America; 3 Cardiovascular Research Institute, University of California San Francisco, San Francisco, California, United States of America; 4 Department of Medicine, University of California San Francisco, San Francisco, California, United States of America; Innsbruck Medical University, AUSTRIA

## Abstract

The final step of triacylglycerol synthesis is catalyzed by acyl-CoA:diacylglycerol acyltransferases (DGATs). We have previously shown that *ApoE*^*-/-*^*Dgat1*^*-/-*^ mice are protected from developing atherosclerosis in association with reduced foam cell formation. However, the role of DGAT1, specifically in myeloid and other hematopoietic cell types, in determining this protective phenotype is unknown. To address this question, we reconstituted the bone marrow of irradiated *Ldlr*^*–/–*^mice with that from wild-type (*WT→ Ldlr*^*–/–*^) and *Dgat1*^*–/–*^(*Dgat1*^*–/–*^*→ Ldlr*^*–/–*^) donor mice. We noted that DGAT1 in the hematopoietic compartment exerts a sex-specific effect on systemic cholesterol homeostasis. However, both male and female *Dgat1*^*–/–*^*→ Ldlr*^*–/–*^mice had higher circulating neutrophil and lower lymphocyte counts than control mice, suggestive of a classical inflammatory phenotype. Moreover, specifically examining the aortae of these mice revealed that *Dgat1*^*–/–*^*→ Ldlr*^*–/–*^mice have atherosclerotic plaques with increased macrophage content. This increase was coupled to a reduced plaque collagen content, leading to a reduced collagen-to-macrophage ratio. Together, these findings point to a difference in the inflammatory contribution to plaque composition between *Dgat1*^*–/–*^*→ Ldlr*^*–/–*^and control mice. By contrast, DGAT1 deficiency did not affect the transcriptional responses of cultured macrophages to lipoprotein treatment *in vitro*, suggesting that the alterations seen in the plaques of *Dgat1*^*–/–*^*→ Ldlr*^*–/–*^mice *in vivo* do not reflect a cell intrinsic effect of DGAT1 in macrophages. We conclude that although DGAT1 in the hematopoietic compartment does not impact the overall lipid content of atherosclerotic plaques, it exerts reciprocal effects on inflammation and fibrosis, two processes that control plaque vulnerability.

## Introduction

Triacylglycerols (TGs) are the predominant energy storage molecules in mammals. Two acyl-CoA:diacylglycerol acyltransferase (DGAT) enzymes, DGAT1 [1] and DGAT2 [2], catalyze the joining of diacylglycerols (DGs) with fatty acyl-CoAs, which is the final and rate-limiting step of TG synthesis. The role of DGAT2 in mammalian physiology has been difficult to study since DGAT2 deficiency is incompatible with life. Mice lacking DGAT2 die within several hours after birth due to a severe skin barrier defect [3]. In contrast, DGAT1-deficient (*Dgat1*^*–/–*^) mice are viable and resistant to diet-induced obesity [4]. Their relative leanness is associated with increased energy expenditure, physical activity [4], non-shivering thermogenesis [5], improved insulin and leptin sensitivity [6, 7], and reduced hepatic steatosis [8]. Ultimately, *Dgat1*^*–/–*^mice have increased life span [9]. Many beneficial effects of DGAT1 deficiency can be reproduced by pharmacologically inhibiting its activity [8, 10–13].

Exploring how DGAT1 deficiency reduces adiposity has highlighted its role in dietary fat assimilation [10–15]. For example, although reducing TG synthesis capacity globally might be expected to increase the systemic and peripheral tissue levels of glycerolipid precursors [DGs and free fatty acids (FFAs)] or metabolites (e.g. ceramides), this does not occur in *Dgat1*^*–/–*^mice [6]. Rather, *Dgat1*^*–/–*^mice have reduced intestinal lipid absorption and an impaired ability of enterocytes to form TGs from dietary FFAs [12, 16]. Moreover, expressing DGAT1 exclusively in the intestine is sufficient to reverse the protection against obesity and hepatic steatosis, normally seen in *Dgat1*^*–/–*^mice fed a diet high in fat [16]. Together, these findings support the concept that the net impact of DGAT1 deficiency on lipid homeostasis is predominantly due to DGAT1 deficiency in enterocytes [13, 16].

In addition to its role in controlling TG homeostasis, DGAT1 may also play a role in atherosclerosis, a disease usually linked to dysregulated cholesterol homeostasis. *Dgat1*^*–/–*^mice bred onto an apolipoprotein E (ApoE)-deficient background have reduced atherosclerotic plaque formation [17]. This reduced lesion size is associated with decreased cholesterol uptake and absorption by the intestine, reduced plasma TG and cholesterol concentrations, and increased cholesterol efflux from macrophages [17]. One possible mechanism for the atheroprotection seen in *ApoE*^*–/–*^*Dgat1*^*–/–*^mice may, at least in part, be akin to the one that drives the metabolic protection seen in *Dgat1*^*–/–*^mice. Specifically, both models may benefit from reduced rates of intestinal lipid uptake and absorption and a suppression of systemic lipid accumulation.

However, while atherosclerosis is traditionally associated with dysfunctional lipid metabolism, considerable evidence has accumulated to implicate vascular inflammation as a key component in atherogenic plaque progression, with activated macrophages residing in the vascular wall acting as key players. Specifically, macrophages engorged with intracellular cholesteryl esters (CEs), or “foam cells”, accumulate in atherosclerotic plaques [18]. Foam cells undergo a form of inflammatory activation, a pathological hallmark of atherogenesis.

With this in mind, it is intriguing to note that, unlike the phenotype of *Dgat1*^*–/–*^mice, DGAT1 deficiency that is limited to tissues and cells other than the intestine has deleterious consequences. In particular, DGAT1 deficiency increases the vulnerability of isolated skeletal muscle to lipotoxicity [19] and potentiates a remarkable exaggeration in the inflammatory response of cultured macrophages to pro-inflammatory lipids [20].

Thus, whereas global DGAT1 deficiency may protect against both plaque development and plaque inflammation in atherosclerosis-prone mice, DGAT1 deficiency specifically in the myeloid compartment might paradoxically worsen atherosclerosis. To test this, we reconstituted the bone marrow of low-density lipoprotein receptor (*Ldlr*^*–/–*^) mice with that of *WT* or *Dgat1*^*–/–*^mice. Our studies show that DGAT1 expression in macrophages has no impact on the lipid content of atherosclerotic plaques developing in response to the consumption of a diet rich in saturated fat and cholesterol, but instead regulates the extent to which plaques exhibit markers of inflammation and fibrosis, two processes that determine plaque vulnerability.

## Materials and Methods

### Animals and diets

*Dgat1*^*–/–*^[4], *WT*, and *Ldlr−*^*/–*^(Jackson Laboratory, Bar Harbor, ME) were on a C57BL/6J background (>10 generations). Animals were housed in a clean barrier facility with regular 12-hour light-dark cycling and had unlimited access to water and either of two standard chow diets [5053 PicoLab (Purina, St. Louis, MO) or Altromin (Lage, Germany)]. All mouse experiments were approved by the Institutional Animal Care and Use Committees (IACUC) at the University of California San Francisco or Medical University of Graz followed either NIH guidelines (AN111420-01C) or those of the Austrian Ministry for Science, Research, and Economy, Division of Genetic Engineering and Animal Experiments (Vienna, Austria) (BMWF-66.010/0057-II/3b/2011) as appropriate.

### Bone marrow transplantation

Eight week-old male and female *Ldlr−*^*/–*^mice were irradiated twice with 5 Gy 3 hours apart 1 day before injection. Bone marrow cells were harvested from age- and sex-matched *WT* and *Dgat1*^*–/–*^donor mice, re-suspended in PBS containing 0.1% FA-free BSA, and intravenously injected (1–1.2 × 10^6^ cells) into the irradiated *Ldlr−*^*/–*^recipient mice. Recipient mice were then treated with antibiotics (Polymyxin B and Neomycin) in water for 4 weeks. Genotyping from blood was performed to confirm bone marrow transplantation efficiency. At 16 weeks of age, the mice were challenged with a Western-type diet (WTD; TD.88137; Harlan-Teklad, IN; containing 21.2% fat, and 0.2% cholesterol by weight) for additional 13 weeks (females) or 19 weeks (males).

### Body composition

Body composition was analyzed by dual energy X-ray absorptiometry (DEXA) with a PIXImus2 scanner (GE Healthcare Lunar, Madison, WI).

### Complete blood cell count

One hundred μl of blood was collected into EDTA-coated tubes (Sarstedt AG, Nuembrecht, Germany). A complete blood cell count was performed using a Hemavet 850 (Drew Scientific Group, Waterbury, CT).

### Plasma lipid parameters

Circulating lipid concentrations were determined from plasma of mice fasted for 10 hours using spectrophotometric kits (TG, Thermo Scientific, Middletown, VA; total cholesterol (TC) and free cholesterol (FC), WAKO chemicals, Richmond, VA). Cholesteryl ester (CE) levels were calculated as the difference between TC and FC.

### Preparation of histological sections for atheroassays

Mice were anesthetized by injection with 2,2,2-tribromoethanol (400 mg/kg) i.p. and then perfused with 1 mM EDTA in PBS through a left-heart intracardiac cannula. Thereafter, mice were perfused with 10% methanol-free formalin for 15 min. Adventitial adipose tissue was carefully removed from the upper part of the aorta, which was then excised from the thoracic cavity, bi-valved in a Y-formation, and stored in 10% methanol-free formalin until staining. The cranial two-thirds of the heart were fixed with 10% methanol-free formalin for 24 hours and then stored in 30% sucrose. Prior to sectioning, the hearts were transferred into Tissue Tek (Sakura Finetek, Torrance, CA), and the aortic roots were serially cryosectioned (8 μm) 24 hours later at -20°C.

### Oil red O (ORO) staining of aortae and aortic valve sections

ORO (0.5 g) was dissolved in 100 ml isopropanol under constant stirring for 24 hours, mixed at a 3:2 ratio with dH_2_O for 10 minutes to create a working solution, which was then filtered and used freshly.

Bi-valved aortae were washed with PBS for 1 min, placed in 70% isopropanol for 5 min, and then stained with the ORO working solution for 20 minutes, after which they were dipped in 70% isopropanol to remove excessive ORO. Stained aortae were pinned out and imaged using a stereo zoom microscope (Leica MZ FL III equipped with Leica DC500 camera).

Aortic root sections were fixed in 10% methanol-free formalin for 10 minutes, placed in 70% isopropanol for 5 more minutes, and then placed in ORO working solution for 15 minutes. Sections were then dipped in 70% isopropanol, counterstained with hematoxylin for 5 minutes, and washed in 0.1% NaHCO_3_. Sections were air dried and mounted with glycerol-gelatin aqueous slide mounting medium (Sigma-Aldrich, St. Louis, MO). Mean lesion area (μm^2^) was calculated from consecutive ORO-stained sections. Images were taken with a Leica DM 4000B microscope equipped with a Leica DFC 500 camera. Plaque areas were quantitated using ImageJ software.

### Immunostaining with monoclonal antibody to macrophages-2 (MoMa-2)

Aortic root sections were fixed for 15 minutes in 10% methanol-free formalin and washed three times with PBS. Sections were then blocked for 30 minutes to protect against endogenous tissue peroxidase activity (Signet Covance, Prineceton, NJ), washed twice with PBS, and then blocked with Ultra V protein block (Lab Vision, Fremont, CA) for 7 minutes. The sections were next incubated in Ultra V protein block solution (1:4 in PBS) containing monoclonal antibody to macrophages-2 (MoMa-2, 1:600; Acris, Hiddenhausen, Germany) for 1 hour at RT, and then overnight at 4°C.

The sections were next washed twice with PBS, incubated for 3 hours with an HRP-coupled polyclonal rabbit anti-rat secondary antibody (1:100, Dako Denmark A/S, Glostrup, Denmark), and washed three times with PBS. AEC substrate (Vector Laboratories, Burlingame, CA) was added until reaction development and then washed away 3 times with H_2_O. Sections were counterstained with hematoxylin for 5 min, washed in 0.1% NaHCO_3_, and mounted with glycerol-gelatin aqueous slide mounting medium (Sigma-Aldrich, St. Louis, MO). Images were taken with a Leica DM 4000B microscope equipped with a Leica DFC 500 camera. The intensity and extent of MoMa-2 staining of three consecutive sections per mouse was quantified using ImageJ software.

### Masson’s Trichrome staining

Slides were incubated in Bouin’s solution (Sigma-Aldrich, St. Louis, MO) overnight at RT, washed under running tap water until the yellow-colored sections became clear, and then washed with dH_2_O (1 minute). Sections were next stained for 3 minutes with a freshly prepared working Accustain® Wigert’s Iron Hematoxylin solution (Sigma-Aldrich, St. Louis, MO), washed first with running tap water for 5 minutes, and then with dH_2_O for 1 minute.

The sections were then stained with Masson’s Trichrome (Sigma-Aldrich, St. Louis, MO) according to manufacturer’s instructions, washed with dH_2_O, and dehydrated (90, 95, and 100% ethanol/xylene). Sections were fixed with Cytoseal-60 mounting medium (Thermo Scientific, Kalamazoo, MI). Images were taken with a ScanScope T3 whole slide scanner (Aperio Technologies, Bristol, UK). Both the blue-stained collagen-rich areas and the acellular necrotic core areas of vascular plaques from three consecutive sections per mouse were quantitated with ImageJ software.

### Isolation and cultivation of peritoneal macrophages

Mice were injected i.p. with 2.5 ml of 3% thioglycollate broth. Their peritoneal cavities were flushed 72 hours later with 10 ml PBS containing 1 mM EDTA (PBS/EDTA). The peritoneal cells were centrifuged and pelleted cells were plated in DMEM (Gibco, Invitrogen, Carlsbad, CA) containing 10% lipoprotein-deficient serum (LPDS) and 1% penicillin/streptomycin (P/S) for 2–3 hours. The plated cells were then washed twice with pre-warmed PBS, and the adherent cells (macrophages) were cultured in DMEM containing 25 mM glucose, 4 mM glutamine, 1 mM pyruvate, 10% LPDS, and 1% P/S for 24 hours. These cells were washed twice with pre-warmed PBS and cultured for additional 24 hours either in the absence or presence of VLDL, acetylated (ac)LDL (both 100 μg protein/ml medium), or LPS as a control (10 ng/ml medium).

### RNA isolation and quantitative real-time PCR analysis

Macrophage RNA was isolated using TriFast™ reagent according to the manufacturer's protocol (Peqlab, Erlangen, Germany). One μg of total RNA was reverse transcribed using the High Capacity cDNA Reverse Transcription Kit (Applied Biosystems, Carlsbad, CA). Quantitative real-time PCR (qPCR) was performed on a Roche LightCycler 480 (Roche Diagnostics, Palo Alto, CA) using the QuantifastTM SYBR® Green PCR kit (Qiagen, Hilden, Germany). mRNA levels were analyzed in duplicate and normalized to those of the reference gene hypoxanthine phosphoribosyltransferase (*Hprt*). Fold-changes and associated statistical parameters were determined by the 2^-ΔΔCT^ method. The following primer sequences were used:

*Arg1*: Fwd 5′-TGGCTTGCGAGACGTAGAC-3′; Rev 5′-GCTCAGGTGAATCGGCCTTTT-3′

*Ccl5*: Fwd 5′-GCTGCTTTGCCTACCTCTCC-3′; Rev 5′-TCGAGTGACAAACACGACTGC-3′

*Gro1*: Fwd 5′-CTGGGATTCACCTCAAGAACATC-3′; Rev 5′-CAGGGTCAAGGCAAGCCTC-3′

*Hprt*: Fwd 5′-TCAGTCAACGGGGGACATAAA-3′; Rev 5′-GGGGCTGTACTGCTTAACCAG-3′

*Nos2*: Fwd 5′-GTTCTCAGCCCAACAATACAAGA-3′; Rev 5′-GTGGACGGGTCGATGTCAC-3′

*Itgax*: Fwd 5′-CTGGATAGCCTTTCTTCTGCTG-3′; Rev 5′-GCACACTGTGTCCGAACTCA-3′

Mcp1: Fwd 5′-ACTGAAGCCAGCTCTCTCTTCCTC-3′; Rev 5′-TTCCTTCTTGGGGTCAGCACA-3′

*Mrc1*: Fwd 5′-GCTGAATCCCAGAAATTCCGC-3′; Rev 5′-ATCACAGGCATACAGGGTGAC-3′

*Tnfa*: Fwd 5′-CCCTCACACTCAGATCATCTTCT-3′; Rev 5′-GCTACGACGTGGGCTACAG-3′

### Statistics

Statistical analyses were performed using GraphPad Prism 5.0 software. Significances were determined using the Student’s unpaired *t*-test and the Welch test (in cases of unequal variances) for two-group comparisons, and ANOVA followed by Bonferroni correction for multiple group comparisons. Data are presented as mean values ± SEM. Significance levels were set at p < 0.05 (*), p ≤ 0.01 (**) and p ≤ 0.001 (***).

## Results

### Bone marrow reconstitution of *WT→* *Ldlr*^*–/–*^and *Dgat1*^*–/–*^*→* *Ldlr*^*–/–*^mice

We investigated the consequences of hematopoietic DGAT1 deficiency on atherogenesis by transplanting *Ldlr*^*–/–*^mice with bone marrow cells from age- and sex-matched *WT* and *Dgat1*^*–/–*^donor mice. PCR analyses of the genomic DNA from peripheral blood cells of transplant recipient mice following recovery confirmed both successful irradiation and the expected genetic chimerism (S1 Fig). Specifically, the donor-derived circulating leukocytes in recipient mice expressed *Ldlr* (S1A Fig), whereas *Dgat1* mRNA in these leukocytes was appropriately present or absent depending on the donor genotype (S1B Fig). We placed both male and female transplant recipient mice on a Western-type high-fat diet (WTD). Since female mice, at least on an *ApoE*^*–/–*^background, develop atherosclerosis more rapidly than males [21], we fed female transplant recipients a WTD for 13 weeks and male recipients a WTD for 19 weeks.

### Comparable body weights and composition of *WT→* *Ldlr*^*–/–*^and *Dgat1*^*–/–*^*→* *Ldlr*^*–/–*^mice

**Fig 1:**
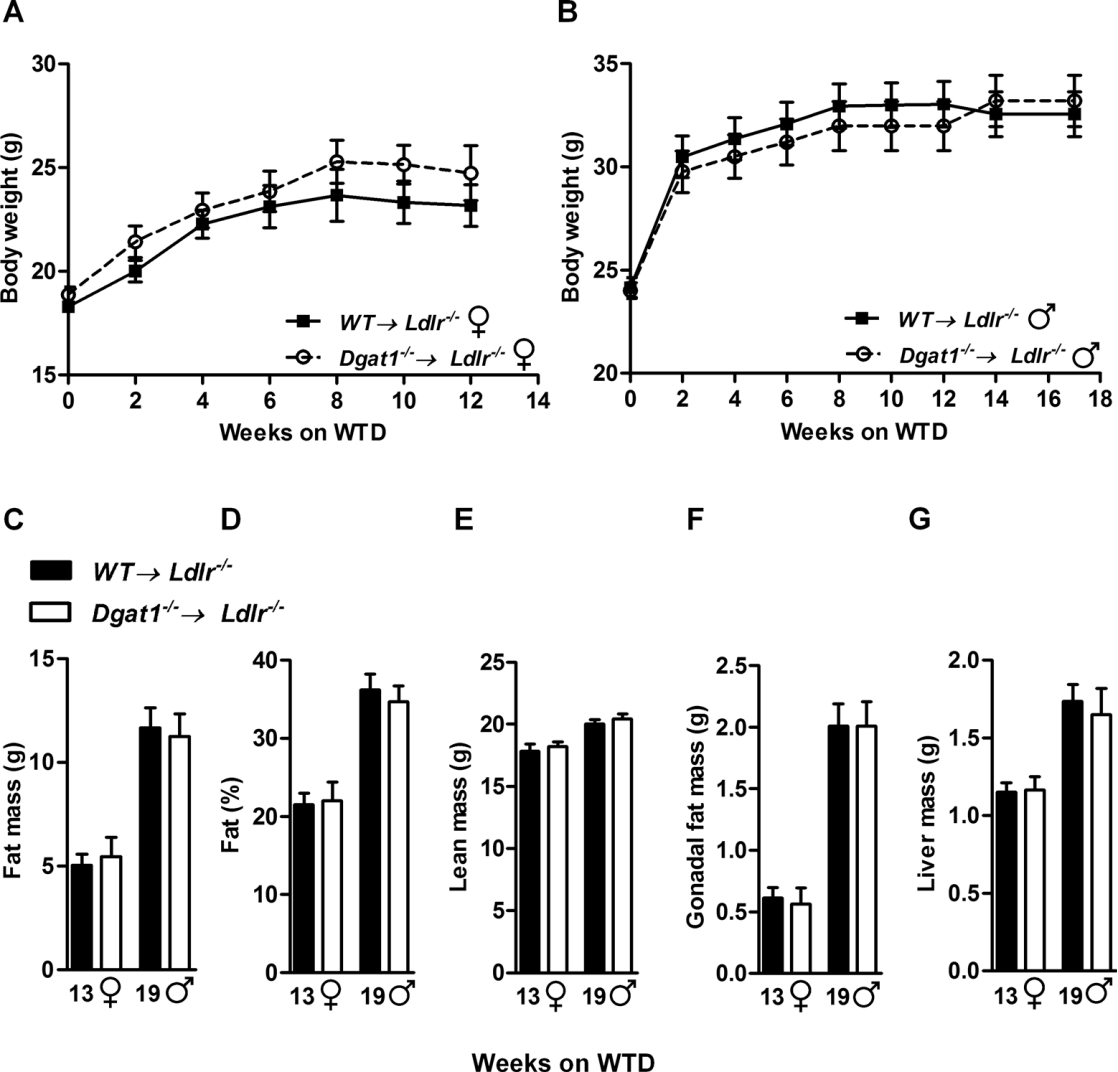
*Dgat1*^*–/–*^*→ Ldlr*^*–/–*^and *WT→ Ldlr*^*–/–*^mice have comparable body weight gain and adiposity. Weight gain of (A) female mice (n = 9) and (B) male mice (n = 10–11) fed a WTD. (C) Total body fat, (D) relative adiposity, (E) lean mass, (F) gonadal fat pad weights, and (G) liver weights of *WT→ Ldlr*^*-/-*^ and *Dgat1*^*-/-*^*→ Ldlr*^*-/-*^ mice after 13 (n = 9 female mice, ♀) and 19 weeks (n = 10–11 male mice, ♂) of WTD, respectively. For all panels, data are presented as means ± SEM.

*Dgat1*^*–/–*^*→ Ldlr*^*–/–*^mice fed a WTD gained a similar amount of weight as controls over both 13 weeks (Fig 1A) and 19 weeks (Fig 1B). Body composition was analyzed one week before the extent of atherosclerosis was analyzed. Dual energy X-ray absorptiometry (DEXA) revealed comparable total body fat (Fig 1C), percent body fat (Fig 1D), and lean mass (Fig 1E) between *WT→ Ldlr*^*–/–*^and *Dgat1*^*–/–*^*→ Ldlr*^*–/–*^mice. The weights of the gonadal fat pads (Fig 1F) and livers (Fig 1G) were also comparable between both genotypes.

### *Dgat1*^*–/–*^*→* *Ldlr*^*–/–*^mice have sex-specific alterations in cholesterol homeostasis

*WT→ Ldlr*^*–/–*^and *Dgat1*^*–/–*^*→ Ldlr*^*–/–*^mice had similar fasting plasma TG concentrations, regardless of sex, after consuming a WTD for 13 weeks (females) or 19 weeks (males). By contrast, deleting DGAT1 from the hematopoietic compartment had a sex-specific impact on systemic cholesterol levels. Female *Dgat1*^*–/–*^*→ Ldlr*^*–/–*^mice fed a WTD (13 weeks) had lower plasma TC concentrations than controls, whereas male *Dgat1*^*–/–*^*→ Ldlr*^*–/–*^mice fed the same diet (19 weeks) had increased plasma TC concentrations (Table 1). Moreover, the effect of hematopoietic DGAT1 deficiency on plasma TC in either sex was coincident with a significant and directionally concordant effect on plasma CE concentration (Table 1). Therefore DGAT1 deficiency among hematopoietic cell types impacts cholesterol homeostasis by altering circulating CE levels, an effect the directionality of which is determined by sex.

**Table 1:** Plasma parameters.

	13 weeks (female mice)		19 weeks (male mice)	
	*WT→ Ldlr*^*–/–*^	*Dgat1*^*–/–*^*→ Ldlr*^*–/–*^	*WT→ Ldlr*^*–/–*^	*Dgat1*^*–/–*^*→ Ldlr*^*–/–*^
**Triglycerides (mg/dl)**	352±45.8	282±24.4	297±22.4	355±27.7
**Total cholesterol (mg/dl)**	1140±76.1	915±45.7*	1409±66.9	1695±54.3**
**Free cholesterol (mg/dl)**	282±17.8	260±13.1	473±33.3	550±35.6
**Cholesteryl esters (mg/dl)**	858±60.1	655±35.4*	935±38	1146±27.8***

Plasma parameters measured after a 10-hour fast in *WT→ Ldlr*^*–/–*^and *Dgat1*^*–/–*^*→ Ldlr*^*–/–*^mice (n = 9–11) chronically fed a WTD. Data are means ± SEM.

*, p< 0.05

**, p ≤ 0.01

***, p ≤ 0.001.

### Altered relative distribution of white blood cell populations in *Dgat1*^*–/–*^*→* *Ldlr*^*–/–*^mice

**Fig 2:**
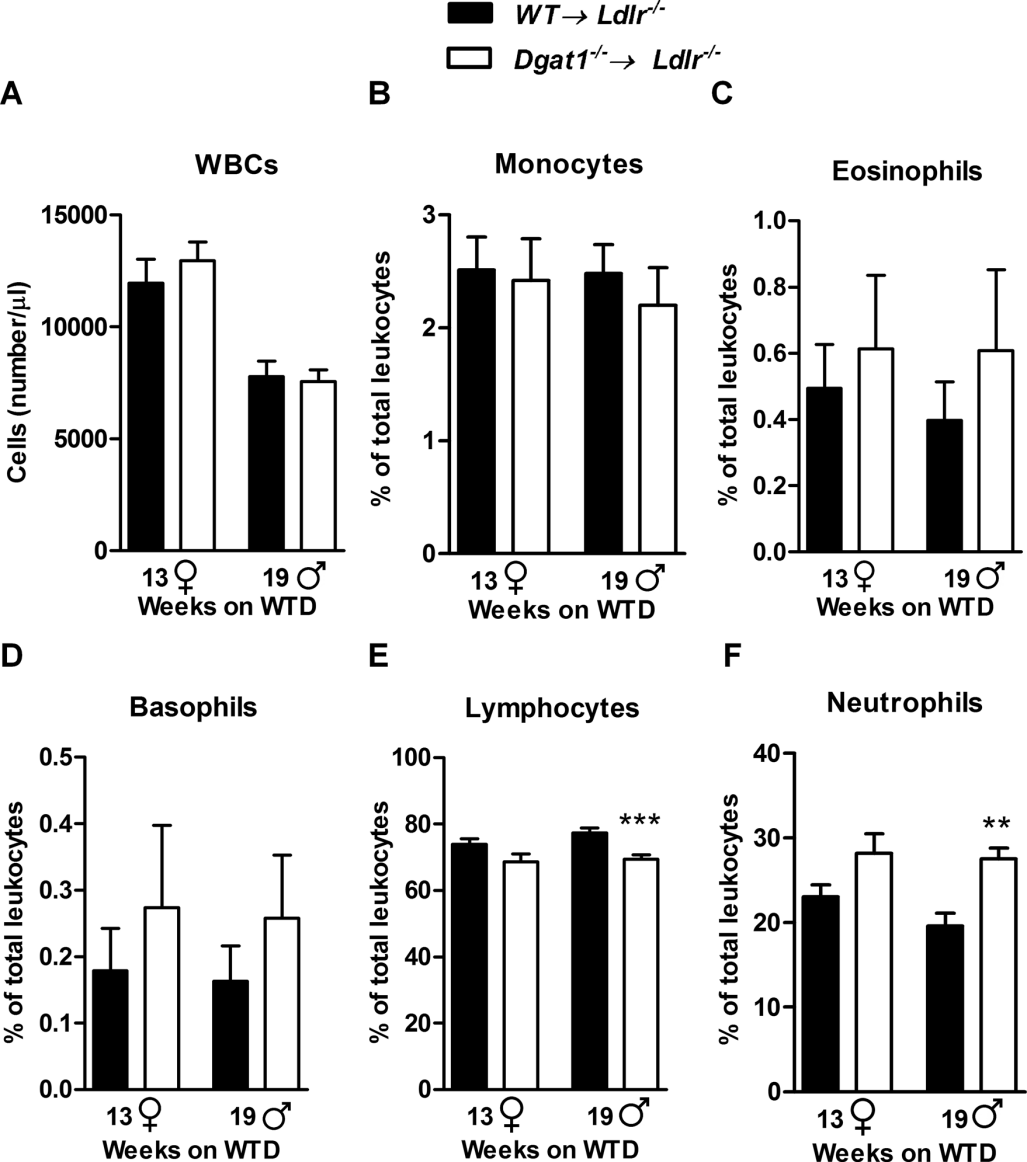
Altered circulating immune cell numbers in *Dgat1*^*–/–*^*→ Ldlr*^*–/–*^mice fed a WTD. (A) Total leukocyte counts, and relative (B) monocyte, (C) eosinophil, (D) basophil, (E) lymphocyte, and (F) neutrophil counts in both *WT→ Ldlr*^*–/–*^and *Dgat1*^*–/–*^*→ Ldlr*^*–/–*^after 13 weeks (females, ♀) and 19 weeks (males, ♂) of WTD feeding (n = 9 per group), showing reduced lymphocyte and increased neutrophil counts in male mice, with a similar trend in female mice. Data are presented as mean ± SEM. **, p ≤ 0.01; ***, p ≤ 0.001.

Since only the hematopoietic system lacked DGAT1 in our mouse model, we monitored the impact of this deficiency on the numbers of white blood cells (WBCs) and their specific subsets in the circulation. The total number of circulating WBCs in both male and female mice was comparable between *WT→ Ldlr*^*–/–*^and *Dgat1*^*–/–*^*→ Ldlr*^*–/–*^mice fed a WTD (Fig 2A), as were circulating levels of monocytes, eosinophils, and basophils (Fig 2B–2D). Lymphocyte frequencies, however, were reduced in both female (trend) and male *Dgat1*^*–/–*^*→ Ldlr*^*–/–*^mice fed a WTD (Fig 2E). By contrast, circulating neutrophil counts were increased in both female (trend) and male *Dgat1*^*–/–*^*→ Ldlr*^*–/–*^mice (Fig 2F). These findings are consistent with the presence of classical systemic inflammation in *Dgat1*^*–/–*^*→ Ldlr*^*–/–*^animals.

### *Dgat1*^*–/–*^*→* *Ldlr*^*–/–*^mice develop pro-inflammatory atherosclerotic plaques

**Fig 3:**
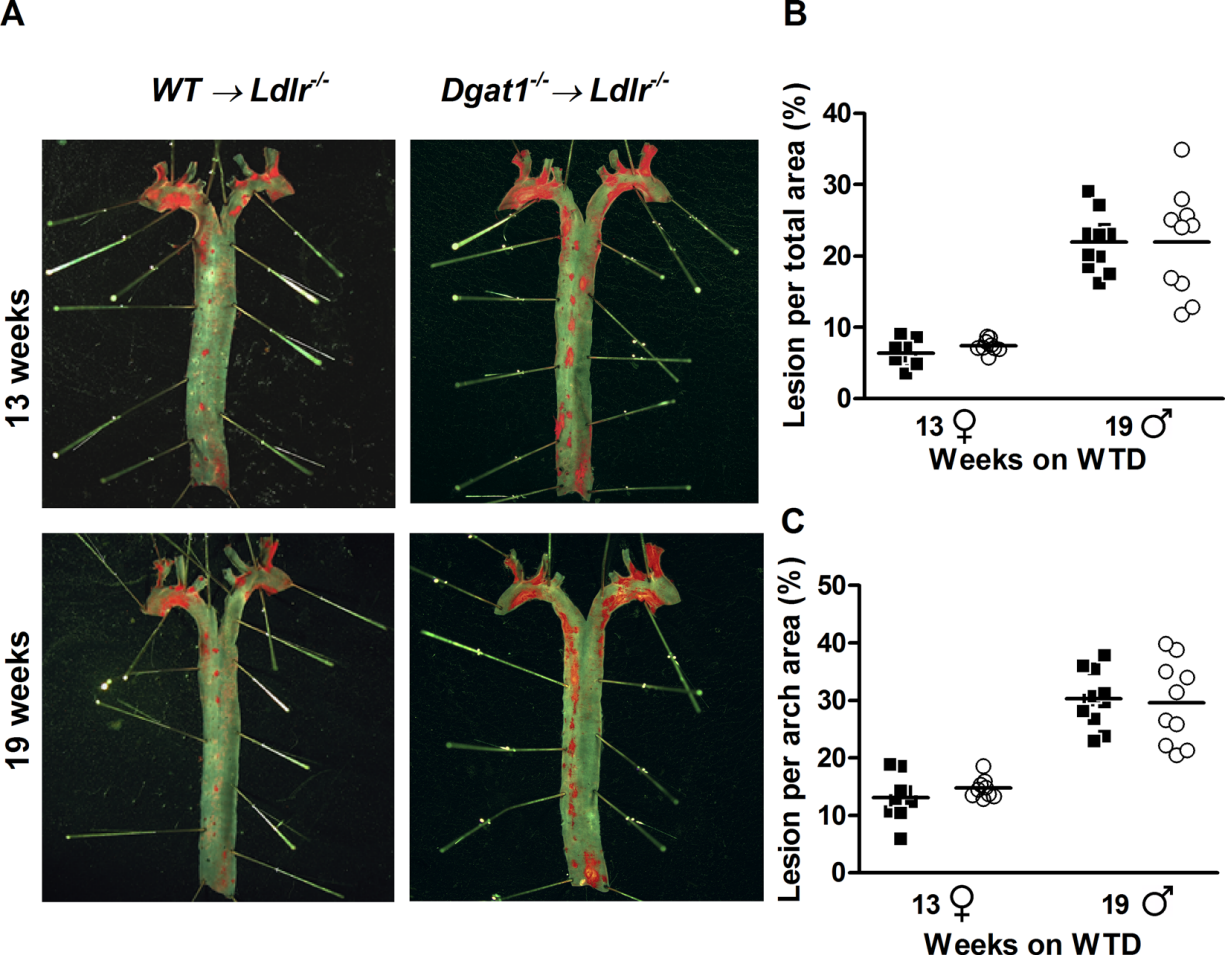
Comparable atherosclerotic plaque areas in the aortas of *WT→ Ldlr*^*–/–*^and *Dgat1*^*–/–*^*→ Ldlr*^*–/–*^mice. (A) Aortae stained *en face* with ORO after 13 and 19 weeks of WTD feeding. (B) Quantification of plaque size in the thoracic aortae and (C) aortic arches of *WT→ Ldlr*^*–/–*^(black squares) and *Dgat1*^*–/–*^*→ Ldlr*^*–/–*^(white circles) mice fed a WTD as in A (n = 9 females, ♀, and 10–11 males, ♂). Horizontal lines represent mean values through data groupings.

**Fig 4:**
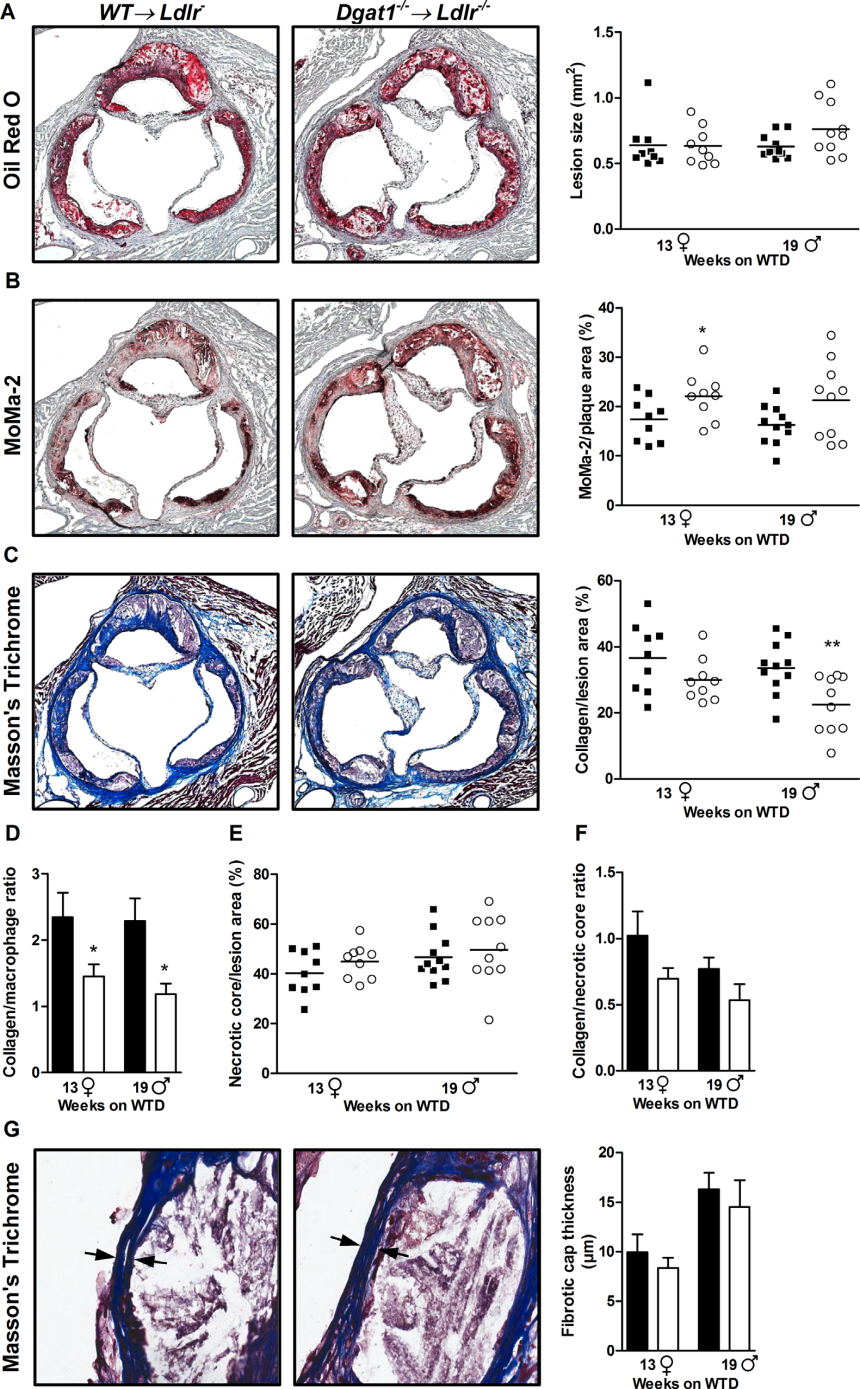
Increased plaque inflammation in *Dgat1*^*–/–*^*→ Ldlr*^*–/–*^mice. (A-C, left) Representative images of aortic root sections stained with ORO, MoMa-2, and Masson's Trichrome. (A-C, right) Quantification of these images to measure plaque size, macrophage content, and collagen deposition, respectively. (D-F) Integrated quantification of data from A-C to determine collagen-to-macrophage ratios, necrotic core per plaque area, and collagen-to-necrotic core ratios, respectively. (G) Representative images of Masson’s Trichrome-stained aortic root sections focused on plaque fibrotic caps, with quantification of minimal fibrotic cap thickness. All data represent means ± SEM of 12 aortic root sections for ORO and three aortic root sections in the area of maximal plaque size for MoMa-2- and Trichrome-staining per mouse after 13 (n = 9 females, ♀) and 19 weeks (n = 10–11 males, ♂) of WTD feeding. *, p < 0.05.

Next, we analyzed atherosclerotic plaque formation along the aortae and in the aortic roots of bone marrow-transplanted animals. *En face* staining revealed that *Dgat1*^*–/–*^*→ Ldlr*^*–/–*^and control mice have comparable atherosclerotic plaque areas in both the thoracic aorta (Fig 3A and 3B) and the aortic arch (Fig 3A and 3C), irrespective of sex. Analyzing the neutral lipid content of aortic root sections showed that *Dgat1*^*–/–*^*→ Ldlr*^*–/–*^and control mice have similarly sized plaques in the aortic valve region as well (Fig 4A).

However, a more detailed analysis of atherosclerotic lesions in both genotypes of mice showed that lesional macrophage content in female *Dgat1*^*–/–*^*→ Ldlr*^*–/–*^mice is greater than control, a finding also seen as a trend in male *Dgat1*^*–/–*^*→ Ldlr*^*–/–*^mice (Fig 4B). This indicates that limiting the ability of hematopoietic cells to synthesize TG promotes innate inflammation in the vascular wall, which is where excess lipid deposition occurs in *Ldlr*^*–/–*^mice. Moreover, lesional collagen content was reduced in male *Dgat1*^*–/–*^*→ Ldlr*^*–/–*^mice and trended similarly in female *Dgat1*^*–/–*^*→ Ldlr*^*–/–*^mice (Fig 4C). Together, these data indicate that atherosclerotic lesions in the aortic roots of *Dgat1*^*–/–*^*→ Ldlr*^*–/–*^mice may be less stable than in controls. This concept was supported by our finding that aortic root plaques from *Dgat1*^*–/–*^*→ Ldlr*^*–/–*^mice have a reduced ratio of collagen to macrophage content, an important indicator of plaque instability (Fig 4D).

On the other hand, other mechanistic determinants of plaque stability were intact despite the loss of DGAT1 among macrophages. For example, the extent of intra-plaque necrosis, defined as acellular compartments within atherosclerotic lesions, was similar across genotypes, irrespective of sex (Fig 4E). The ratio of plaque collagen to intra-plaque necrosis in aortic roots was also comparable between genotypes (Fig 4F). Similarly, minimal thicknesses of plaque fibrotic caps, which correlates with the risk of plaque rupture and intraluminal thrombosis, was comparable between *Dgat1*^*–/–*^*→ Ldlr*^*–/–*^and control mice (Fig 4G). Thus, plaque structures were largely similar between both genotypes, suggesting that the differences in plaque composition between these genotypes is limited to their inflammatory nature.

### Macrophage phenotype is regulated independently of DGAT1 expression

**Fig 5:**
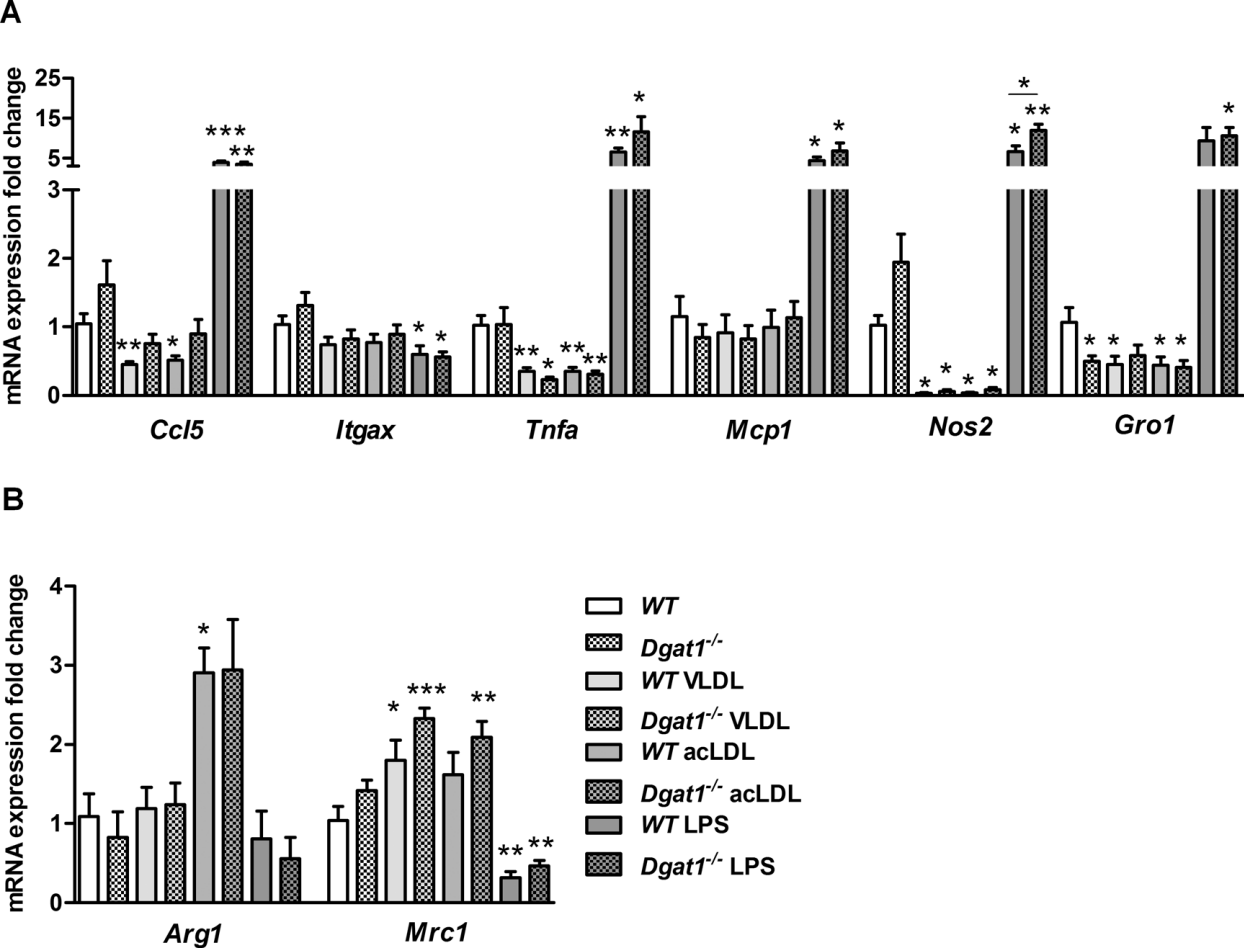
M_1_ and M_2_ gene expression in lipoprotein-treated *WT* and *Dgat1*^*–/–*^macrophages. qPCR analysis of control macrophages vs. those treated with 100 μg/ml VLDL, 100 μg/ml acLDL or 100 ng/ml LPS (positive control for M_1_ activation). Shown are mRNA levels of (A) M_1_ marker and (B) M_2_ marker genes analyzed in duplicate. Data are means (n = 3–6) ± SEM. *, p < 0.05; **, p ≤ 0.01; ***, p ≤ 0.001.

Based on our *in vivo* findings, we sought to determine if altering DGAT1-dependent lipid handling by macrophages impacts inflammatory responsiveness at a cell-intrinsic level. Therefore, we investigated the effects of lipoprotein loading on the polarization state of elicited mouse peritoneal macrophages *in vitro*. As expected, treating these macrophages for 24 hours with LPS induced a classical inflammatory transcriptional response, and thus served as a positive control. On the other hand, the mRNA levels of genes marking both the classical M_1_ (*Ccl5*, *Itgax*, *Tnfa*, *Mcp1*, *Nos2*, and *Gro1*) (Fig 5A) and alternative M_2_ (*Arg1* and *Mrc1*) (Fig 5B) macrophage polarization states were comparable between *Dgat1*^*–/–*^and *WT* macrophages, whether untreated or loaded with VLDL, acLDL, or LPS. These results indicate that DGAT1 deficiency does not heighten the inflammatory responsiveness of macrophages to lipoprotein stress in this model, a distinction from the vulnerability to inflammatory activation that was reported for *Dgat1*^*–/–*^macrophages treated with free pro-inflammatory saturated FAs [20].

## Discussion

Numerous reports have shown that DGAT1 deficiency and pharmacological inhibition have beneficial effects on whole body metabolism, lipid homeostasis, and longevity [4–13]. Our previous finding that global *ApoE*^*–/–*^*Dgat1*^*–/–*^mice have reduced atherosclerotic plaque formation [17] left us wondering whether amelioration of the atherosclerosis in this model is a consequence of reduced intestinal cholesterol uptake and absorption, resulting in lower plasma cholesterol levels, or alterations in macrophage function.

To address this, we adoptively transferred *WT* and *Dgat1*^*–/–*^hematopoietic cells, respectively, into irradiated *Ldlr*^*–/–*^recipient mice. We chose *Ldlr*^*–/–*^over *ApoE*^*-/-*^ mice as recipients since in contrast to *Ldlr*^*–/–*^mice, transplantation of ApoE-expressing bone marrow into *Apoe*^*-/-*^ recipients is able to repair hyperlipidemia and atherosclerosis [22, 23]. Our approach reveals that DGAT1 expression specifically in the hematopoietic compartment limits the inflammatory impact of WTD consumption on vascular plaques in the context of LDL receptor deficiency while having little if any effect on atherosclerosis *per se* or on other structural determinants of plaque composition. The effect of hematopoietic DGAT1 deficiency on increasing plaque macrophage content in the aortic root was mirrored by a relative systemic neutrophilia and lymphocytopenia, both markers of classical inflammation. Finally, it was interesting to note that mice lacking DGAT1 in immune cells had alterations in circulating CE and TC levels, indicative of a role for these cells in regulating systemic cholesterol homeostasis.

DGAT1 has been well demonstrated to exert effects on energy balance and body weight. For example, both genetically deleting DGAT1 throughout the body in mice and pharmacologically inhibiting its activity systemically leads to a reduction in body weight and adiposity [4, 12, 13]. By contrast, overexpressing DGAT1 in the white adipose tissue of mice leads to increased adiposity and a propensity for diet-induced obesity [20]. Macrophages are immunological sentries and coordinators of tissue remodelling and homeostasis throughout the body that notably also express DGAT1. Here we assessed the effect of DGAT1 deficiency in the hematopoietic compartment, which gives rise to macrophages, on energy homeostasis, and found that it does not alter body weight gain, adiposity, or liver size in mice fed a WTD. This finding indicates that DGAT1 deficiency limited to immune and other hematopoietic cells is insufficient to alter whole body energy homeostasis. Our results mirror those seen in mice overexpressing DGAT1 specifically in macrophages, a model that also fails to impact body weight and adiposity [20].

In contrast to the lack of effect on fat storage, we found that *Dgat1*^*–/–*^*→ Ldlr*^*–/–*^mice had alterations in systemic lipid homeostasis. Specifically, hematopoietic DGAT1 deficiency impacted fasting plasma TC and CE levels versus *WT→ Ldlr*^*–/–*^controls. The intriguing aspect of this finding, however, is that the effects were directionally opposite in male and female mice. Whether this discrepancy arises from sex-specific differences in how macrophages regulate cholesterol homeostasis or whether differences in the duration of the WTD (13 weeks in females, 19 weeks in males) reveal a temporally bi-phasic effect on cholesterol homeostasis in our model remains unclear. Regardless, it is important to point out that the changes we saw in systemic cholesterol levels reflect the capacity of DGAT1, expressed exclusively in the hematopoietic compartment, to determine systemic cholesterol levels. This finding will be followed up in future studies.

Bone marrow transplantation exerts specific long-term effects on hematopoiesis in recipients [24]. Given this, we were encouraged to find that total WBC numbers were similar in mice receiving *Dgat1*^*–/–*^and *WT* bone marrow in our study, indicating that DGAT1 is not required for normal hematopoiesis. This finding corroborates what is already known about global *Dgat1*^*–/–*^mice, which do not have any gross hematopoietic defect. Given this, it was interesting to note that *Dgat1*^*–/–*^*→ Ldlr*^*–/–*^mice in our study did have alterations in the relative abundance of specific circulating WBC populations following chronic consumption of a WTD. Both male and female mice tended to have elevated circulating neutrophil counts and concomitantly reduced lymphocyte counts. Since neutrophils have been implicated in promoting the pathogenesis of atherosclerosis [25] by modulating processes associated with the vulnerability and inflammatory properties of vascular plaques [26, 27], it is reasonable to speculate that neutrophils may have influenced the plaque phenotype in *Dgat1*^*–/–*^*→ Ldlr*^*–/–*^mice or that their systemic levels reflect a response to chronically increased plaque inflammation.

When quantified by monitoring overall neutral lipid content, *WT→ Ldlr*^*–/–*^and *Dgat1*^*–/–*^*→ Ldlr*^*–/–*^mice had a similar aortic plaque distribution and area, regardless of sex. We found that aortic valve lesions developed more rapidly in female mice (13 weeks of WTD) than in males (19 weeks of WTD), and these sex-specific differences were not impacted by DGAT1 deficiency in the hematopoietic compartment.

Although it is used to track plaque growth, the neutral lipid content of atherosclerotic plaques may not be a clinically valuable pathological marker of plaque advancement. On the other hand, the number and polarization state of macrophages within atherosclerotic plaques have been implicated in mediating the advancement of these lesions [28]. Interestingly, the key effect of hematopoietic DGAT1 deletion on plaque structure in the female mice we studied here was to visibly increase the number of intra-plaque macrophages, a trend that was also seen in male mice.

Enzymes involved in lipid metabolism and handling within the hematopoietic compartment have been shown to be important in systemic lipid homeostasis and atherosclerosis [29–35]. However, because such enzymes have potentially pleiotropic roles in systemic metabolism, the results of deleting genes encoding them in mice often have surprising effects on atherosclerosis.

For example, lipoprotein lipase (LPL) deficiency in the bone marrow might be expected to impair intravascular TG hydrolysis. However, such mice do not have altered systemic TG levels, and unexpectedly display reduced systemic TC concentrations. Indeed, hematopoietic LPL deficiency is associated with reductions in both macrophage ApoE secretion andatherosclerosis [29]. Similarly, mice lacking the intracellular TG hydrolytic enzyme adipose triglyceride lipase (ATGL) in the bone marrow have reduced atherosclerotic progression, despite the prediction that ATGL-deficient macrophages would accumulate TG and thus aggravate foam cell formation and atherogenesis [36]. By contrast, macrophages deficient in peroxisome proliferator-activated receptor γ (PPARγ) are resistant to foam cell formation, yet display increased inflammatory potential and a concomitant vulnerability to atherogenesis [37].

It was thus unclear what impact hematopoietic DGAT1 deficiency would have on plaque inflammation in *Dgat1*^*–/–*^*→ Ldlr*^*–/–*^mice, and our observation that these mice have an increased plaque macrophage burden has several potential mechanistic implications. First, it underscores the concept that enhancing the capacity of macrophages to stably store intracellular fatty acids (FAs) within TG is anti-inflammatory. It also suggests that increasing macrophage TG storage capacity limits the flux of intracellular FAs into pro-inflammatory species. These could include glycerolipid intermediates such as lysophospholipids and DGs, and lipids of alternate metabolic pathways, including sphingolipids, ceramides, and phospholipids.

We also investigated the inflammatory responsiveness of cultured *Dgat1*^*–/–*^macrophages. We have previously shown that directly exposing macrophages to saturated FAs drives *Dgat1*^*–/–*^macrophages to a pro-inflammatory, M_1_-like phenotype [20], which is associated with complicated, destabilized atherosclerotic lesions [38]. However, the mechanism by which saturated FAs exert their pro-inflammatory effects is an area of ongoing investigation, as is the nature of the enhanced inflammatory vulnerability of *Dgat1*^*–/–*^macrophages.

More recently, we have shown that the flux of intracellular FAs into the phospholipid compartment of macrophages mediates activation of the NLRP3 inflammasome [39]. This suggests that at least one way how DGAT1 deficiency contributes to a pro-inflammatory predisposition may be through limiting the flux of intracellular FAs into TG, and increasing their flux into phospholipids. Membrane phospholipids are important in the release of arachidonic acid, a precursor of eicosanoid synthesis [40]. Therefore, increased phospholipid flux in macrophages could affect systemic immune responsiveness by altering eicosanoid homeostasis.

Unlike in metabolic tissues such as the WAT, skeletal muscle, and liver, where the concentration of FAs and their metabolites may rise in the context of diet-induced obesity and exert pro-inflammatory effects on resident and infiltrating macrophages, lipid-activation of macrophages in atherosclerotic plaques may be driven more by locally deposited LDL, including species with pro-atherogenic modifications. Given this, it isinteresting that unlike the response we saw in macrophages treated with FFAs [20], the M_1_ response of macrophages treated with either VLDL or acLDL was not impacted by the presence or absence of DGAT1. This finding suggests that the role DGAT1 plays in the manner by which macrophages handle FFAs taken up from their environment is different from the role it plays in macrophage handling of intracellular FAs during the course of lipoprotein uptake and metabolism.

It should be noted, however, that the current study used macrophages that were elicited into the peritoneum by thioglycollate, harvested and plated, and then studied in culture. Such elicited peritoneal macrophages change their polarization from more naïve M_0_-like state to a “pre-activated” M_1_-like state. Therefore, these macrophages may have already been somewhat primed prior to lipoprotein or LPS treatment, and this could have limited our ability to measure the pro-inflammatory effects of VLDL or acLDL treatments in both *WT* and *Dgat1*^*–/–*^macrophages. On the other hand, LPS-treatment of peritoneal macrophages in our study robustly induced the transcription of M_1_ genes, indicating that these cells are still amenable to further activation by a pro-inflammatory stimulus.

The collagen content and collagen-to-macrophage ratios of plaque lesions were reduced in *Dgat1*^*–/–*^*→ Ldlr*^*–/–*^animals, whereas the thickness of the fibrotic cap, which protects the lesion from rupture and the necrotic core from leakage into arterial lumen, was comparable between both genotypes. These results indicate that at least some indices marking plaque instability are worsened when macrophages lack DGAT1 in the context of progressive atherosclerosis in mice. It will be important to discern what factors reduce collagen deposition in this model while sparing cap fibrotic potential. Both *WT→ Ldlr*^*–/–*^and *Dgat1*^*–/–*^*→ Ldlr*^*–/–*^plaques contained a high proportion of intra-plaque necrosis, commonly found in advanced lesions [41]. This is not surprising, as plaque inflammation and downstream necrosis may have been potentiated in both groups of mice by exposure to ionizing irradiation in preparation for bone marrow transplantation [42–44].

In summary, we examined the contribution of macrophage and other hematopoietic DGAT1 expression on atherosclerosis by studying *Ldlr*^*–/–*^mice reconstituted with bone marrow taken from *Dgat1*^*–/–*^mice. We found that the resulting *Dgat1*^*–/–*^*→ Ldlr*^*–/–*^mice developed atherosclerotic lesions with increased macrophage content and showed a tradeoff between reduced plaque collagen content and greater inflammation. However, hematopoietic DGAT1 deficiency had no appreciable impact on overall plaque burden or other aspects of plaque structure (lipid content, necrosis, cap thickness).

## Conclusion

Our data support the possibility that DGAT1 plays a dual role in atherosclerosis. On one hand, global DGAT1 deficiency protects against atherosclerosis [17]. On the other hand, we show here that DGAT1 in macrophages may specifically participate in limiting the development of plaque inflammation. Our findings strongly suggest that the former finding in *ApoE*^*–/–*^*Dgat1*^*–/–*^mice is due to DGAT1 deletion in another cell or tissue type. One possibility is that DGAT1 deficiency in the gut reduces that rate at which dietary TG and cholesterol is assimilated, thus limiting the availability of the lipid substrate for plaque development in *ApoE*^*–/–*^*Dgat1*^*–/–*^mice.

## Supporting Information

S1 Fig Successful bone marrow transplantation from *WT* and *Dgat1*^*-/-*^ donors to *Ldlr*^*-/-*^ recipient mice. (PDF)
