# Supplementary material for: Acyl-CoA:Diacylglycerol Acyltransferase 1 Expression Level in the Hematopoietic Compartment Impacts Inflammation in the Vascular Plaques of Atherosclerotic Mice
Source: PLoS One. 2016 May 25;11(5):e0156364. doi: 10.1371/journal.pone.0156364 (PMC4880185; doi:10.1371/journal.pone.0156364)
Supplement: S1 Fig — (PDF) [file pone.0156364.s001.pdf]

SUPPLEMENTAL MATERIAL

Supplemental Figure and Figure Legend

Supplemental Figure S1

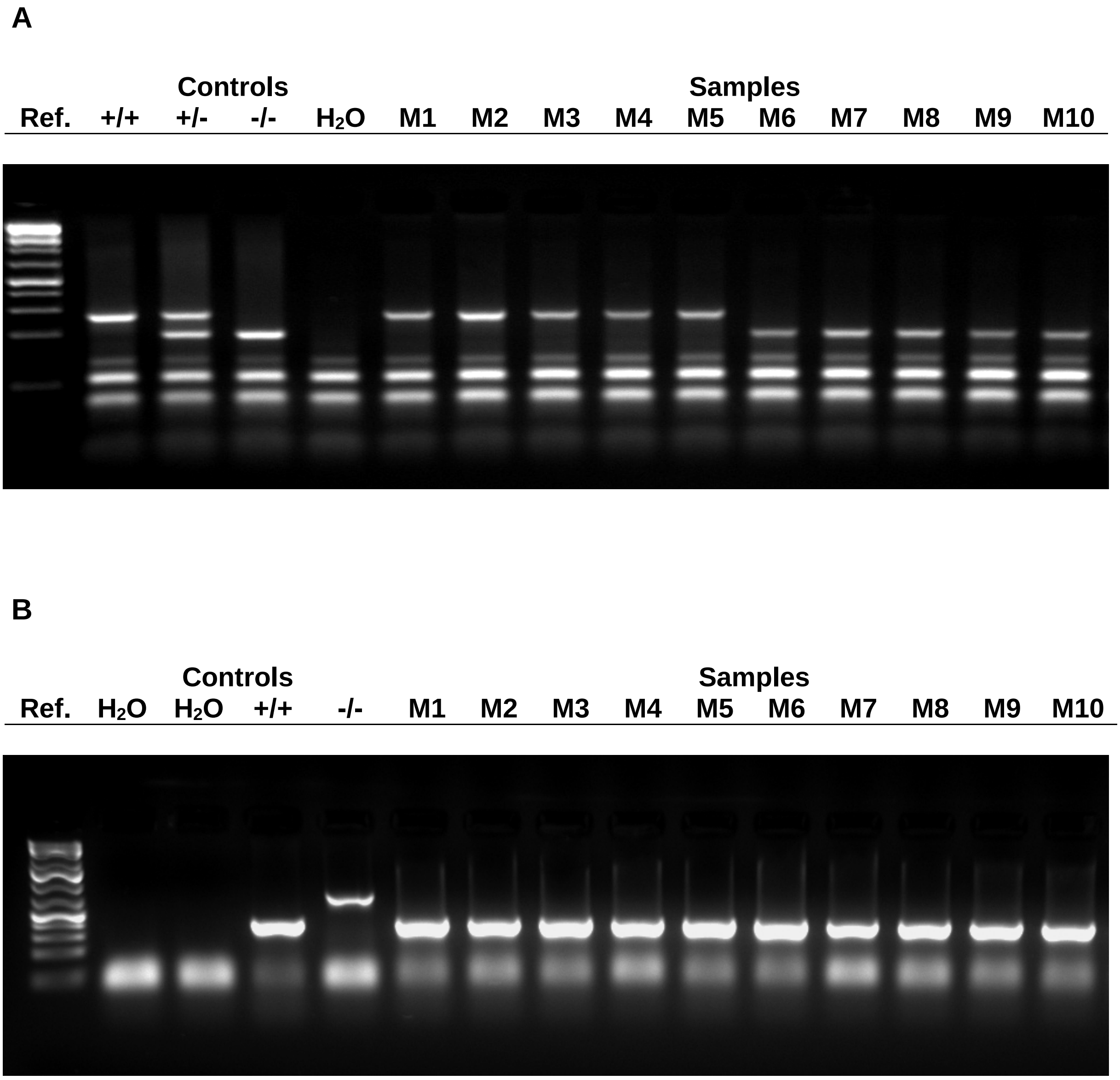

Fig S1. Successful bone marrow transplantation from *WT* and *Dgat1$^{-/-}$* donors to *Ldlr$^{-/-}$* recipient mice. Four weeks post-transplantation, peripheral blood samples were used for (A) Dgat1 and (B) Ldlr genotyping. PCR products were analyzed by agarose gel electrophoresis. Images show representative gels for mice transplanted with *WT* or *Dgat1$^{-/-}$* bone marrow, respectively.
